# Initial experiences of the Optima™ coil system in intracranial aneurysm treatment: surgical and interventional approach to safety and efficacy in terms of cerebral arteries

**DOI:** 10.1590/acb403425

**Published:** 2025-05-09

**Authors:** Bekir Sıtkı Said Ulusoy, Çetin Murat Altay, Mehmet Onay, Ali Burak Binboga, Murat Kaya

**Affiliations:** 1 University of Health Sciences – Gaziantep City Hospital – Department of Interventional Radiology – Gaziantep – Turkey.

**Keywords:** Intracranial Aneurysm, Endovascular Procedures, Angiography, Cerebral Arteries, Embolization, Therapeutic

## Abstract

**Purpose:**

To evaluate the angiographic outcomes of the Optima™ coil system in the endovascular treatment of saccular intracranial aneurysms to present real-world experiences.

**Methods:**

The study encompassed patients with both ruptured and unruptured aneurysms who underwent treatment with the Optima™ coil system. A retrospective analysis was conducted to examine patient and aneurysm characteristics, complication rates, and angiographic outcomes.

**Results:**

The total of 326 Optima™ coil implantations was performed in 64 aneurysms, with a mean maximum diameter of 7.49 ± 3.08 mm (range: 2.5–16.5 mm), among 64 patients (37 females and 27 males, mean age: 53.34 ± 14 years old). The average number of implanted coils was 5.06 ± 1.73. The mean packing density was 26.2% (range = 19.2–34.6), observed to be significantly higher in aneurysms with complete occlusion and neck remnants compared to those with a residual dome (p < 0.01). The mean follow-up period was 15.9 ± 8.1 months. One case (1.5%) reported a coil malfunction attributed to coil stretching. The mortality rate was 3.1% (n = 2).

**Conclusion:**

The Optima™ coil system exhibited safety and efficacy in the endovascular treatment of both ruptured and unruptured intracranial aneurysms, demonstrating favorable angiographic outcomes. Nevertheless, further studies are necessary to validate these results over the long term.

## Introduction

Endovascular treatment of intracranial aneurysms has revolutionized the management of these potentially life-threatening vascular abnormalities. It offers a minimally invasive alternative to open surgical approaches, such as craniotomy and aneurysm clipping. In addition to being minimally invasive, endovascular treatment also has the bonus of reduced recovery time and less trauma to surrounding tissue. This treatment modality involves navigating a catheter through the vascular system to the site of the aneurysm, allowing for interventions aimed at preventing rupture or treating an already ruptured aneurysm. With advances in technology and technique, endovascular treatment has shown promising long-term outcomes in preventing aneurysm rupture. The decision between endovascular therapy and open surgery depends on the aneurysm’s size, shape, and location, as well as patient-specific factors such as age, health status, and personal preferences. Regular follow-up imaging, such as magnetic resonance imagings or computed tomography scans, is necessary after endovascular treatment to monitor the stability of the treated aneurysm^1^^–^ 3.

The first detachable coil designed for the endovascular treatment (EVT) of intracranial aneurysms (IAs) was pioneered by Guido Guglielmi^4^^,^ 5. Since then, the approach to IA treatment through coil embolization has steadily advanced. The advantages of endovascular coiling are emphasized in evidence-based guidelines for the EVT of IAs^6^. With progress in this field, coiling has emerged as the predominant treatment choice, indicating a paradigm shift in numerous cases^7^. However, the coiling process may present challenges in specific cases, primarily influenced by the morphological characteristics of the aneurysm^8^. The success of the EVT procedure is not solely contingent on aneurysm morphology; it is also significantly affected by endovascular tools, including coil properties and others.

As authors, we are aware that flow diversion device and emerging stent technologies constitute current and popular topics in interventional neuroradiology. Nevertheless, we contend that coil embolization, a pivotal component of endovascular aneurysm treatment, and innovative coil technologies should not be disregarded. Detachable coils have undergone rapid development, surpassing their predecessors in sophistication. Newly manufactured coils introduce new features that enhance the coiling process, making it more effective and less risky.

In this context, one of these innovative coils, the Optima^TM^ coil system (Balt LLC, United States of America), has recently become available in many countries, claiming to possess cutting-edge features. The Optima™ Coil System is a cutting-edge device used for the EVT of IAs. It belongs to a class of detachable platinum coils designed to be inserted into the aneurysm sac to induce clotting and prevent the aneurysm from filling with blood, thereby reducing the risk of rupture. The system provides advanced features that enhance procedural safety, efficiency, and effectiveness in treating both ruptured and unruptured aneurysms. The manufacturer claims that the Optima^TM^ coil system incorporates features that improve the efficiency and safety of EVT, such as a progressively softer push wire, an open-loop coil configuration, a small and soft first loop, a very short detachment time, and a reduced number of detachment attempts. To date, there is one poster presentation^9^ and two ongoing trials to further document the safety and efficacy of the treatment of IAs with the Optima coil system^10^^,^ 11. In the poster presentation by Abdelsalam et al., they stated that they did not encounter any complications or adverse events related to Optima or OptiMAX coils in a single center case series of 20 patients^9^. To date, no study has been conducted at the literature level that examines the above-mentioned Optima™ coil system reflecting real-world experience.

This cohort study aimed to evaluate the angiographic outcomes of the Optima^TM^ coil system in the EVT of saccular IAs to present real-world experiences.

## Methods

This retrospective study was approved by the local institutional review board/ethics committee (protocol no.: 242).

### Patients and aneurysms

Patients who underwent EVT for IAs, both ruptured and unruptured, between January 2019 and January 2023, were included in the study. Medical records were obtained from an interventional radiology database and retrospectively reviewed. Aneurysm status (ruptured and unruptured), aneurysm maximum diameter (Dmax), aneurysm neck length, dome-neck ratio (D/N), and aneurysm localization were noted from preoperative diagnostic digital subtraction angiography (DSA) images. Glasgow Coma Scale (GCS) and modified Rankin Scale (mRS)^12^ were utilized to determine the patients’ neurological status. mRS 0-2 indicated favorable clinical outcomes, while mRS 3-4 indicated poor clinical outcomes.

### Angiographic data

The Raymond-Roy occlusion classification (RROC) was used to determine the aneurysm occlusion grade immediately and at follow-up. It is an angiographic classification scheme for grading the occlusion of endovascularly treated intracranial aneurysms^13^. It is also known as the Raymond class, Montreal scale, or the Raymond Montreal scale:

- Class I: complete obliteration;
- Class II: residual neck;
- Class III: residual aneurysm.

The scheme was originally created to evaluate aneurysm occlusion class, not predict aneurysmal recurrence. Though not truly equivalent, these are often equated to aneurysmal obliteration of 100, > 90, and < 90%, respectively.

### Modified Raymond-Roy classification

Mascitelli et al.13 in 2015 proposed a modified Raymond–Roy classification (MRRC) or modified Montreal scale, in which class III is subdivided to reflect progression to occlusion:

- Class IIIa: contrast opacification with the coil interstices of a residual aneurysm;
- Class IIIb: contrast opacification outside the coil interstices, along the residual aneurysm wall.

**Figure 1:**
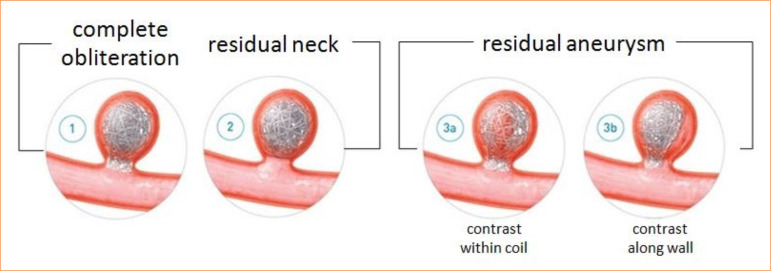
Schematic view of Raymond-Roy classification.

Source: Elaborated by the authors.

The study from Mascitelli et al.^13^ found that class IIIa aneurysms progress to complete occlusion more often than class IIIb aneurysms. A validation study by Stapleton et al.^14^ confirmed that the predictive capability of the RROC was improved by the MRRC, showing not only that IIIa occluded more often (53.6 *versus* 19.2%), but that IIIb lesions would also further recanalize more frequently (65.1 *versus* 27.4%) (Fig. 1).

Aneurysm occlusion grades were obtained in a consensual manner by two interventional neuroradiologists with at least five years’ experience. The RROC 1 and 2 were considered as satisfactory aneurysm occlusion grades. The number of implanted coils was reviewed bearing in mind the patient records. Implanted coil volume (CV) was calculated through Eq. 1:


$$\mathrm{CV}\;=\;\pi {\left(P:2\right)}^{2}\;L$$ (1)

where: P = primary coil diameter; L = coil length, in mm.

Aneurysm volume (AV) was calculated using software (Syngo Aneurysm Guidance Neuro VD 20, 2012, Siemens Healthcare, Illinois, United States of America).

Packing density (PD) (%) was calculated through Eq. 2:


PD = (CV:AV) × 100 (2)

Coil malfunctions were specified as a failure of the coil to detach, coil fracture during deployment, and coil stretching. Aneurysm sac rupture, loss of access due to microcatheter kickback during the coiling process, and coil prolapse were defined as coil-related complications. To identify coil malfunctions and coil-related complications, intraoperative DSA images were screened and re-evaluated by the same two interventional neuroradiologists. Complications were evaluated patients using intraoperative DSA images and patient records separately. Patient status and aneurysm characteristics, complication rates, and angiographic outcome data were taken into consideration to investigate the safety and efficacy of Optima^TM^ coil system.

### General endovascular operation procedure

**Figure 2:**
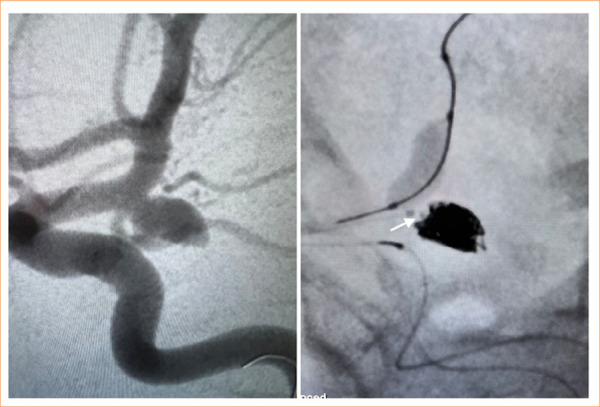
Balloon-assisted coiling utilizing the Optima^TM^ coils in a patient with a ruptured Pcom aneurysm. The white arrow in left indicates the proximal end-tip marker of the Optima^TM^.

Source: Elaborated by the authors.

**Figure 3:**
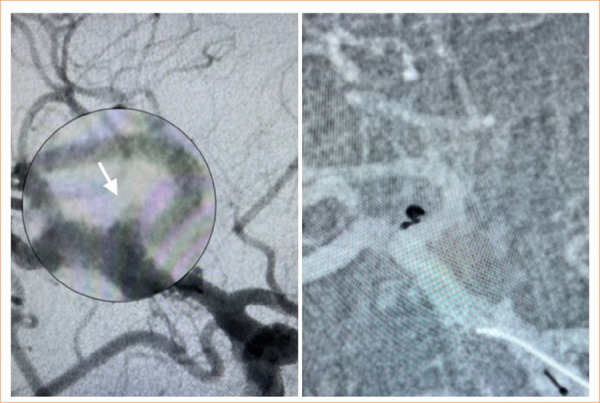
Simple coiling utilizing the Optima^TM^ coil in a patient with a ruptured middle cerebral artery aneurysm. The white arrow on the right indicates the very small aneurysm.

Source: Elaborated by the authors.

All EVT procedures were performed under general anesthesia, including invasive arterial pressure monitoring. A bolus dose of 5,000 IU heparin was administered to achieve systemic anticoagulation. Heparin infusion was initiated to maintain an activated clotting time greater than two times the baseline value (approximately 250–300 seconds). After placing the microcatheter into the aneurysm sac, the coil was loaded. The coil size was determined according to the aneurysm sac diameter. Coiling was performed until adequate angiographic aneurysm occlusion was achieved or no additional coils could be safely added. The EVT was finished after the final control based on DSA images in standard and working projections. Illustrative cases are presented in Figs. 2 and 3.

### Statistical analysis

Data were transferred to the Statistical Package for the Social Sciences v23 (IBM) software for analysis. Study data were expressed as frequency distribution (median, number, percentage, minimum–maximum) for categorical variables and descriptive statistics (mean, standard deviation, range) for numerical variables. χ^2^ and T tests were used to compare two independent data, and *p* < 0.05 was considered significant.

## Results

In 29 patients, Optima^TM^ coils were used in combination with Barricade (Balt LLC, United States of America) and ev3 Axium (Medtronic, Minneapolis, MN, United States of America) coils, as Optima^TM^ coils of appropriate size were not available during the procedure. Stent assisted coiling was performed in 11 patients with unruptured aneurysms. These patients were excluded from the study to specifically highlight the effectiveness of Optima^TM^ coils. Consequently, the real-world outcomes of a total of 326 Optima^TM^ coils in 64 patients treated exclusively with the Optima^TM^ coil system are presented in this study.

### Patients and aneurysm characteristics

Demographic and aneurysm characteristics of the study cohort were summarized in Table 1. Among the 42 patients with subarachnoid hemorrhage (SAH), 59.1% had a preoperative Glasgow Coma Scale (GCS) score of 14–15, 27.3% had a GCS score of 9–13, and 13.6% had a GCS score of 3–8. All none-SAH patients (n = 22) had a mRS score of 0. Acutely ruptured aneurysms were treated in the first 48 hours. The mean Dmax of the aneurysms was 7.49 ± 3.08 mm. The mean neck length and D/N were 3.97 ± 1.36 mm and 1.62 ± 0.18, respectively. The most common aneurysm localization was the anterior communicating artery (n = 25, 39%).

**Table 1:** Baseline characteristics of patients and aneurysms.

Variables	N (%) = 64
Age ± SD years	53.34 ± 14
Sex	37 females and 27 males
Presentation subarachnoid hemorrhage None-subarachnoid hemorrhage	42 (65.6٪) 22 (34.4٪)
**Aneurysms**	
D_max_ ± SD (mm)	7.49 ± 3.08 (range = 2.5–16.5 mm)
Neck length (mm)	3.97 ±1.36 (range = 1.9–8.2 mm)
D/N ± SD	1.62 ± 0.18
**Localization**	
Anterior communicating artery	25 (39%)
Internal carotid artery	20 (31.2%)
Middle cerebral artery	16 (25٪)
Basilar	3 (4.7 ٪)

SD: standard deviation; D_max_: maximum aneurysm diameter; D/N: dome neck ratio; SD: standard deviation D/N. Source: Elaborated by the authors.

### Treatments

Patients were treated with balloon-assisted coiling (BAC) or simple coiling. Table 2 demonstrates treatment features and complications. The intraoperative sufficient aneurysm occlusion grades (RROC 1 and 2) were 92.8%. The mean packing density was 26.2 (minimum–maximum = 19.2–34.6). Subgroup analysis revealed a statistically significant difference in PD between aneurysms with RROC 1 and 2 compared to RROC 3 (*p* < 0.01). There was a positive correlation between PD and aneurysm occlusion.

**Table 2:** Treatment details and angiographic results.

Variables	n (%)
Total Optima^TM^ coil numbers	326
Number of Optima^TM^ coils per patient ± SD	5.06 ± 1.73
Packing density (%)	26.2 (minimum–maximum: 19.2–34.6)
**Endovascular technique**	
Simple coiling	10^nr^ + 28^r^ (59.3)
Balloon-assisted coiling	12^nr^ + 14^r^ (40.6)
**Intraoperative angiographic outcomes**	
RROC 1	26 (40.6)
RROC 2	33 (51.5)
RROC 3	5 (7.8)
**Complications**	
Distal embolism	1 (1.5)
Coil loops prolapse	1 (1.5)
Coil malfunction*	1 (1.5)
Coil migration during follow-up	None
Follow-up (months)	15.9 ± 8.1
Re-treatment rate	6 (9.3)
Mortality	2 (3.1)

SD: standard deviation; RROC: Raymond-Roy occlusion classification; *Coil stretching; r: ruptured aneurysm; nr: non-ruptured aneurysm. Source: Elaborated by the authors.

### Complications

One coil malfunction was observed, it was coil stretching (1.5%). Two (5.6%) complications occurred, one coil-related and one balloon-related. A coil loop prolapsed during BAC in a patient with a ruptured anterior communicating artery aneurysm. Despite balloon manipulations, a stent was deployed to salvage the prolapsed coil loop into the parent artery. The patient did not experience neurological deficits. The second case involved distal emboli in a patient treated for a ruptured right middle cerebral artery aneurysm, resulting in temporary left upper extremity weakness.

### Follow-up

The mean follow-up period was 15.9 ± 8.1 months. Retreatment occurred in 9.3% (n = 6) of cases, with five patients having RROC 3 and one patient with RROC 2 retreated due to aneurysm enlargement. No coil loop migration or aneurysm re-rupture occurred during the follow-up period. The mortality rate was 3.1% (n = 2).

## Discussion

This study presents the results of only patients treated with the Optima^TM^ coil system. It provides clinical evidence supporting the use of Optima^TM^ coils in the EVT of both ruptured and unruptured IAs. Notably, we observed minimal coil malfunction and coil-related complication, high aneurysm occlusion rates, and low morbidity and mortality rates.

Advances in tools and techniques have enhanced the feasibility of EVT with simple or assisted coil embolization being the preferred and standardized method for IAs^7^^,^ 15. Coils implanted in the aneurysm sac induce intrasaccular thrombosis, minimizing the risk of aneurysm rupture^16^^,^ 17. Besides patient and aneurysm characteristics, the physical properties of implanted coils significantly influence the coiling process. Coil stiffness, shape memory, frictional force in the microcatheter, stability in pulsatile flow, detachment duration, and the number of attempts are critical factors^18^. Coil stiffness and shape memory are pivotal in deploying the inherent tridimensional configuration of the coil, thereby preventing excessive microcatheter kickback during detachment, which could lead to the loss of access to the aneurysm sac. Daniel et al.^18^ emphasized that loss of access is a crucial complication of coil embolization.

Additionally, in challenging aneurysm morphologies, renavigation into the aneurysm sac may prove impossible, and multiple renavigation attempts can cause intimal damage, potentially resulting in parent artery thrombosis^18^^–^ 20. Soft coils have the potential to decrease the failure rate by minimizing kickback severity^20^. We did not note microcatheter prolapse or re-access attempts due to kickback when treating either ruptured or unruptured aneurysms. Aneurysm size significantly affects the safety and effectiveness of EVT^15^. Small aneurysm sizes pose an independent risk for intraprocedural aneurysm rupture, especially during the detachment of the first coil^21^^,^ 22. The design of the initial coil loop and the softness of the coil may also impact intraprocedural aneurysm rupture. Optima^TM^ coils have small and soft initial coil loop. The TARGET Registry reported a 2.7% intraoperative perforation rate associated with TARGET^®^ coils (Stryker Corp., Fremont, California, United States of America) in the management of both ruptured and unruptured IAs with a median size of 5.6 mm^23^. Likewise, the SMART Registry (SMART COIL^®^ System, Penumbra Inc., Alameda, California, United States of America) documented a 2.9% procedural device-related serious adverse event rate with SMART coils in the treatment of ruptured and unruptured IAs, with a mean size of 6.9 mm^24^.

In our study, we observed no intraprocedural ruptures or serious device-related adverse events. However, it is crucial to approach the interpretation of our results with caution regarding the perceived superiority of Optima^TM^ coils, considering the expected reduction in intraoperative complication rates in larger aneurysms and the average aneurysm size of 7.49 mm. It is important to note that our findings do not conclusively establish the superiority of Optima^TM^ coils, underscoring the necessity for further studies.

Aneurysm occlusion grades are linked to the PD of coils and the use of assisted coiling techniques^25^. In order to exclusively evaluate the contribution of coils and PD to aneurysm occlusion, patients who underwent stent-assisted coiling were excluded from the study. This exclusion was necessary due to the impact of stents on altering blood flow, a factor contributing to aneurysm occlusion. PD plays a crucial role in intra-aneurysmal hemodynamics and thrombus formation^25^. In the TARGET Registry and SMART Registry, the median PDs were 28.8 and 32.3%, respectively, while the immediate RROC grades 1–2 were 91.7 and 97.1%^23^^,^ 24. The AMERICA Study with Axium MicroFX ev3 coils (Medtronic; Plymouth, Minnesota, United States of America) reported an 85% adequate occlusion immediately post-procedure, with no available data on PD^26^. We found the median PD and immediate RROC 1–2 rates (26.2 and 92.2%) to be acceptable. Moreover, our study demonstrated a positive correlation between PD and aneurysm occlusion rates. Aneurysms with high PD exhibited high occlusion grades.

Coil detachment properties, especially in BAC, are vital. The manufacturer’s catalog indicates that the Optima^TM^ coil system has an extremely short detachment time, specifically less than 1 second. We observed very fast detachment and low attempts. We value the fast deployment features because, especially in cases in which BAC technique is used, particularly when we need to deploy several coils sequentially before deflating the balloon^27^, there is a potential to reduce the occlusion time in the parent artery, thereby decreasing the rates of distal emboli and related complications^27^^,^ 28. In our observation, the quick detachment time and a low number of detachment attempts may provide the operator with an opportunity to increase the PD with the aneurysm by allowing more coil deployments in the limited time the balloon remains inflated. Comparative studies with other coil systems are warranted for further insights.

One device malfunction was observed. It was coil stretching, and thromboembolic events were minimal. We reported favorable initial angiographic outcomes. The absence of coil loop migration during the six-month follow-up is noteworthy, as coil mass stability is crucial in preventing complications. Device malfunction-related serious adverse events were absent in our study, may reflecting the reliability of the Optima^TM^ coil system.

Despite these promising findings, our study has limitations, including its retrospective, single-centered design, and a relatively small sample size. Nevertheless, our study’s value lies in being the first one to present clinical results and operator experiences with the Optima^TM^ coil system. However, our initial findings need confirmation through larger, multicenter, and randomized studies to establish the long-term efficacy and safety of the Optima^TM^ coil system in the treatment of IAs.

## Conclusion

The Optima^TM^ coil system demonstrated significant promise as an interventional EVT method for IAs. Our findings indicated that it is not only safer but also faster and less complicated compared to conventional surgical approaches. This minimally invasive technique offers a streamlined treatment option that could reduce patient recovery time and procedural risks. Further longitudinal studies are encouraged to confirm its sustained safety and effectiveness.

## Data Availability

All data supporting the findings of this study can be requested from the corresponding author.
